# Spatial and temporal characteristic of PM2.5 and influence factors in the Yellow River Basin

**DOI:** 10.3389/fpubh.2024.1403414

**Published:** 2024-07-31

**Authors:** Li Han, Meng Han, Yiwen Wang, Hua Wang, Jiqiang Niu

**Affiliations:** ^1^School of Computer Science and Technology, Zhengzhou University of Light Industry, Zhengzhou, China; ^2^Key Laboratory for Synergistic Prevention of Water and Soil Environmental Pollution, Xinyang Normal University, Xinyang, China

**Keywords:** PM2.5, Geodetector, driving factors, Yellow River Basin, zoning, spatiotemporal variation

## Abstract

The Yellow River Basin has been instrumental in advancing ecological preservation and fostering national high-quality development. However, since the advent of China’s reform and opening-up policies, the basin has faced severe environmental pollution issues. This study leverages remote sensing data from 1998 to 2019. As per the “Basin Scope and Its Historical Changes” published by the Yellow River Conservancy Commission of the Ministry of Water Resources, the Yellow River Basin is categorized into upstream, midstream, and downstream regions for analysis of their spatial and temporal distribution traits using spatial autocorrelation methods. Additionally, we employed probes to study the effects of 10 factors, including mean surface temperature and air pressure, on PM2.5. The study findings reveal that (1) the annual average concentration of PM2.5 in the Yellow River Basin exhibited a fluctuating trend from 1998 to 2019, initially increasing, then decreasing, followed by another increase before ultimately declining. (2) The air quality in the Yellow River Basin is relatively poor, making it challenging for large-scale areas with low PM2.5 levels to occur. (3) The PM2.5 concentration in the Yellow River Basin exhibits distinct high and low-value concentration areas indicative of air pollution. Low-value areas are predominantly found in the sparsely populated central and southwestern plateau regions of Inner Mongolia, characterized by a better ecological environment. In contrast, high-value areas are prevalent in the inland areas of Northwest China, with poorer natural conditions, as well as densely populated zones with high energy demand and a relatively developed economy. (4) The overall population density in the Yellow River Basin, as well as in the upstream, midstream, and downstream regions, serves as a primary driving factor. (5) The primary drivers in the middle reaches and the entire Yellow River Basin remain consistent, whereas those in the upper and lower reaches have shifted. In the upstream, air pressure emerges as a primary driver of PM2.5, while in the downstream, NDVI and precipitation become the main influencing factors.

## 1 Introduction

Since the reform and opening up, along with the continuous advancement of urbanization and industrialization in China, the atmospheric environment has faced dual pressures from population growth and economic development (1, 2). In 2013, haze blanketed the central and eastern regions of China, affecting 25 provinces and over 100 large and medium-sized cities. This phenomenon has emerged as one of the most critical factors influencing the quality of China’s economic development, public welfare, and residents’ health (3). Research has demonstrated that PM2.5, the primary pollutant exacerbating haze, poses risks to the human respiratory, circulatory, and nervous systems (4, 5). The Chinese government has traditionally placed significant emphasis on ecological and environmental issues. The report of the 19th CPC National Congress emphasizes the importance of addressing pollution in tandem with economic development, listing pollution prevention and control as one of the three major battles to achieve comprehensive prosperity. Therefore, studying the spatial and temporal distribution characteristics and causes of PM2.5 is crucial for mitigating China’s severe air pollution and addressing regional pollution, primarily characterized by PM2.5.

Currently, scholars have engaged in extensive discussions on PM2.5-related issues, resulting in enriched research findings. These research topics primarily involve analyzing the sources and chemical composition (6) of PM2.5, its health effects (7), spatial and temporal characteristics, and influencing factors. Among these topics, studies on the spatial and temporal patterns have established a multi-scale framework covering countries (8–10), urban clusters (11–13), provinces (14–17), and prefectural cities (18–21). Some researchers have also focused on watershed studies; for instance, Wang et al. (22) employed spatial autocorrelation analysis to conclude the stable spatial autocorrelation characteristics of PM2.5 pollution in the Yangtze River Economic Belt; similarly, Pang Q. H. et al. have conducted related research (23). Xu Y. et al. employed descriptive statistics and Markov chain methods to demonstrate the significant seasonality of atmospheric pollution in cities within the Huaihe River Ecological and Economic Belt, revealing considerable spatial distribution disparities (24). Utilizing the hotspot analysis method, it was determined that both cold spot and hot spot areas expanded in the Pearl River Delta region from 2000 to 2020. Most of the aforementioned studies focused on the Yangtze River, Huaihai, and Pearl River basins, revealing significant spatial and temporal variations in PM2.5 distribution and changes due to the vast coverage and varying natural conditions of these basins. Therefore, analyzing PM2.5 pollution in watershed areas holds great significance for advancing ecological civilization and fostering high-quality development.

Researchers worldwide have employed various methods to investigate the spatial and temporal dynamics of PM2.5 and its influencing factors. Spatial and temporal evolution characteristics of PM2.5 are studied using methods such as the spatial center of gravity transfer model (25), hot spot analysis method (26), standard ellipsoid difference model (27), spatial autocorrelation model (11, 28), etc. Spatial autocorrelation analysis, as a spatial statistical method, effectively reveals the spatial structure of variables with spatial distribution characteristics by determining spatial correlations and quantifying their degree. In terms of PM2.5-driven analysis, traditional regression models like the Pearson correlation coefficient (29), system generalized moment estimation (SGMM) (30), grey correlation model (31), and OLS regression are commonly used. Additionally, researchers incorporate spatial perspectives into models, including the spatial Durbin model (32), multiscale geographically-weighted regression model, geographically-weighted regression model (33), spatial metrics model (34), geographic detector model (35, 36), etc. Spatial models, accounting for spatial correlation, adhere more closely to objective laws than traditional models. Particularly, the geographic detector is favored by researchers as a new statistical method to detect spatial variability and reveal driving factors due to its wide applicability and immunity to covariate covariance of multiple independent variables. It identifies the strength of factors’ influence and explores the explanatory power of natural socio-economic factors on PM2.5 variability and its significance (37).

The Yellow River Basin, which stretches across central and eastern China, serves as a vital ecological barrier and economic hub, playing a significant role in nationwide ecological protection and sustainable development (38). In 2019, the Yellow River Basin’s ecological protection and high-quality development were designated as a major national strategy, presenting new goals and requirements for the coordinated advancement of the regional economy, society, and ecological environment. Currently, scholars both domestically and internationally have investigated the primary control elements and influencing mechanisms of PM2.5 pollution in the Yellow River Basin. For instance, Li and Han (39) employed geographic detectors to analyze influencing factors in the Yellow River Basin from 2000 to 2017, offering theoretical insights and decision-making guidance for adapting Yellow River management objectives to diverse spatial patterns based on local conditions. Geng et al. (40) utilized geographically weighted regression to examine influencing factors in the Yellow River Basin during the “13th Five-Year Plan” period. They concluded that population density, the number of industrial enterprises, and land use intensity are the primary socioeconomic factors contributing to the increase in PM2.5 concentration. However, most studies treated the study area as a homogeneous entity, overlooking the inherent socioeconomic disparities among the upper, middle, and lower reaches of the basin. Furthermore, many existing studies in the Yellow River Basin solely rely on cross-sectional data for driver analysis, neglecting the spatial and temporal fluctuations of these drivers.

Existing research has extensively investigated multi-scale research systems, including countries, city clusters, and prefectural cities. However, there remains a dearth of studies focusing on watersheds, particularly in terms of zonal analyses. Moreover, most existing watershed studies rely solely on cross-sectional data for driver analysis, overlooking the spatial and temporal heterogeneity of these drivers. Therefore, this study aims to address these gaps by utilizing annual average PM2.5 concentration data from 1998 to 2019 in the Yellow River basin. The basin will be partitioned according to the Basin Scope and its Historical Changes outlined by the Yellow River Conservancy Commission of the Ministry of Water Resources. This research seeks to analyze the spatial and temporal patterns of PM2.5 changes within the Yellow River basin and its subregions. Additionally, Geodetectors will be employed to investigate the driving mechanisms of PM2.5 at four specific time nodes: 2003, 2008, 2013, and 2019. The findings of this study aim to provide valuable insights and recommendations for the sustainable development of the Yellow River basin.

## 2 Data and methods

### 2.1 Overview of the research area

**Figure 1:**
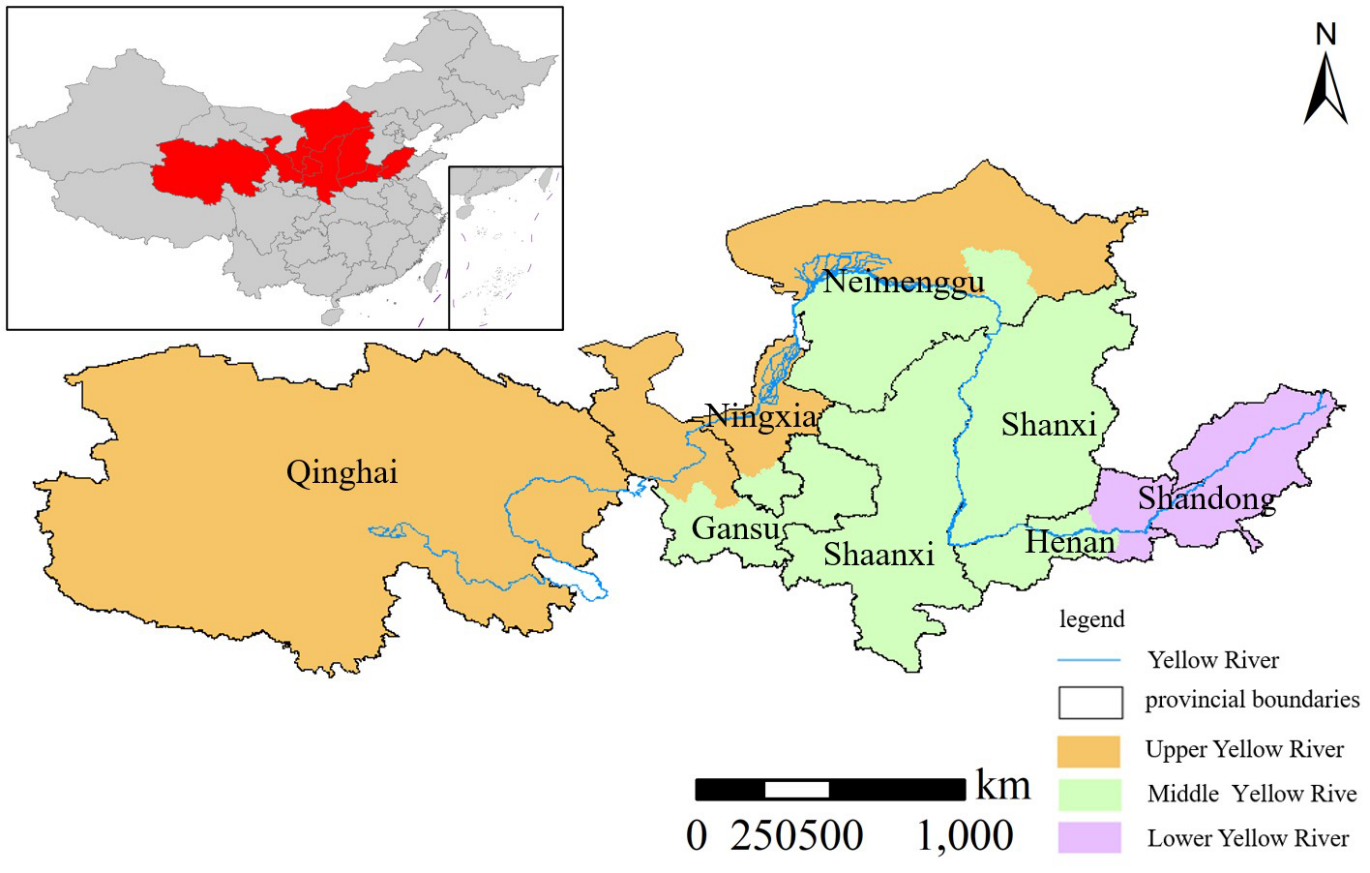
Location map of the Yellow River Basin.

The Yellow River Basin originates from the northern foothills of the Bayan Har Mountains on the Qinghai-Tibet Plateau and traverses through Qinghai Province, Gansu Province, Ningxia Hui Autonomous Region, Inner Mongolia Autonomous Region, Shaanxi Province, Henan Province, and Shandong Province. According to the “Scope of the Basin and its Historical Changes” issued by the Yellow River Conservancy Commission of the Ministry of Water Resources, the upstream of the Yellow River spans from its source area to above Hekou Town in Toketo County of the Inner Mongolia Autonomous Region, mainly situated in the Qinghai-Tibet Plateau and the Ningxia-Henan Loess Plateau, covering an area of 3.65 × 105 km^2^. The middle reaches extend from Hekou Town to Huayuankou in Zhengzhou City, Henan Province, encompassing regions across Shaanxi, Shanxi, Inner Mongolia, Gansu, Ningxia, and Henan, covering an area of 4.08 × 105 km^2^. The lower reaches stretch from Taohuayu to the mouth of the Yellow River, covering Henan and Shandong Provinces, with an area of 0.23 × 105 km^2^. The upstream of the Yellow River Basin is characterized by mountainous terrain, while the lower reaches are characterized by plains and hills. Since the reform and opening up, the adoption of high-emission production methods, intense development, and construction activities, and dense population and industrial layouts have exacerbated the PM2.5 pollution problem in the Yellow River Basin (21). In this paper, considering that the primary tributaries of the Yellow River in Sichuan Province only traverse Aba County, Hongyuan County, Ruoergai County, Songpan County in Aba Prefecture, and Shiqu County in Ganzi Prefecture, and given that Sichuan Province is part of the Yangtze River Economic Belt, while the East Mengdong region (including Hulunbeier, Chifeng, Tongliao, Xing’anmeng, and Xilinguolemeng) in Inner Mongolia falls within the Northeast region, the study area delineated in this paper encompasses counties from eight provinces, excluding Sichuan, and the East Mengdong region in Inner Mongolia (Figure 1).

### 2.2 Data sources

#### 2.2.1 PM2.5 data sources

The remotely sensed PM2.5 raster data inversion was sourced from the Atmospheric Composition Analysis Group at the University of Washington.^1^ This dataset integrates Aerosol Optical Depth (AOD) retrievals from NASA MODIS, MISR, and SeaWiFS instruments with the GEOS-Chem chemical transport model, calibrated with geographically weighted regression (GWR) against global ground-based observations. Spanning from 1998 to 2019, it offers a spatial resolution of approximately 1 km × 1 km. Specifically for China, the product number is V4.CH.03, providing high spatial resolution data that accurately depicts PM2.5’s spatial distribution pattern. Widely adopted in global and regional PM2.5-related studies, this dataset serves as a valuable resource (41–43).

#### 2.2.2 Other data

This study aims to comprehensively investigate the impact of climatic factors, population density, economic activities, and human actions on PM2.5 concentrations. Meteorological variables such as temperature, air pressure, insolation, and precipitation are recognized as pivotal factors influencing PM2.5 levels. Temperature and insolation can modulate chemical reaction rates and the formation of photochemical by-products in the atmosphere, thus influencing both the production and reduction processes of PM2.5. Fluctuations in barometric pressure can alter atmospheric dispersion patterns and pollutant transport dynamics, thereby affecting the spatial distribution and concentration levels of PM2.5. Furthermore, precipitation serves as a natural cleansing mechanism, effectively scavenging particulate matter from the atmosphere and consequently mitigating PM2.5 concentrations.

In addition to meteorological influences, NDVI, serving as a proxy for vegetation cover and health, can significantly impact the deposition and adsorption of particulate matter in the atmosphere. Population density and economic activities, exemplified by the value added of secondary production and local fiscal expenditures, are closely intertwined with industrial operations and transportation activities, serving as notable sources of PM2.5 emissions. Conversely, nocturnal luminosity and electricity consumption indirectly reflect the degree of urbanization and human activity levels, thereby influencing the emission and dispersion patterns of atmospheric pollutants.

**Figure 2:**
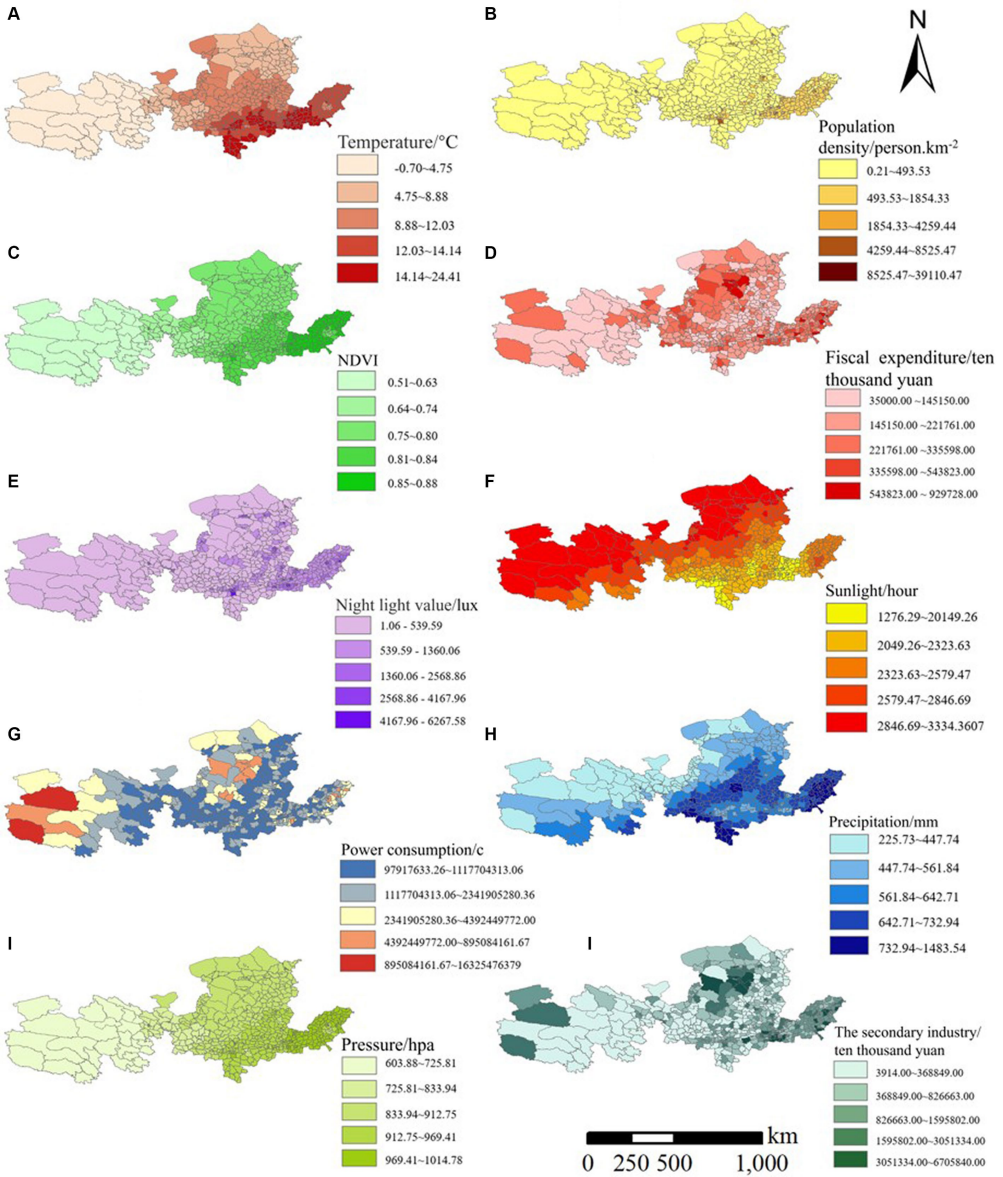
Spatial distribution schematic diagram of explanatory variables.

In this paper, the aforementioned factors are categorized into two main groups: natural and social factors (Figure 2). Natural factors encompass mean surface temperature, mean barometric pressure, sunshine hours, precipitation, and NDVI. Mean surface temperature data is sourced from the China Surface Temperature LST Annual 1KM dataset available on the Resource and Environmental Science Data Registration and Publishing System website.^2^ Mean barometric pressure, sunshine hours, and precipitation data are obtained from the National Platform for Shared Services of Scientific and Technological Resources-National Earth System Science Data Center and the National Earth System Science Data Center.^3^ The vegetation index is derived from the MODIS NDVI product dataset MOD13A3 provided by NASA. Socioeconomic factors include population density, value added to secondary industries, local fiscal expenditures, nighttime lighting, and electricity consumption.^4^ Population density data is acquired from LandScan, a global population dataset by Oak Ridge National Laboratory.^5^ Data on value added to the secondary industry are sourced from the China County Statistical Yearbook. Local fiscal expenditures data is obtained from the Fiscal Revenue and Expenditure Data of Individual Counties (Municipalities and Districts) in China. Data on nighttime lighting is sourced from the China Long Time Series Year-by-Year Artificial Nighttime Lighting Data Set provided by the National Tibetan Plateau Science Data Center.^6^ Electricity consumption data is from a study by Jiandong Chen et al., published in Scientific Data.^7^

### 2.3 Research methodology

#### 2.3.1 Spatial autocorrelation analysis

Spatial autocorrelation refers to the inherent spatial interdependence observed in geography, which helps characterize the relationships between proximal geographic points and temporal variables across different locations. Given the strong spatial coherence of atmospheric phenomena, PM2.5 concentration values exhibit closer proximity spatially. Spatial autocorrelation statistics serve to illustrate the relationships among geographic observations and are commonly employed to examine the spatial clustering and patterns of geographic elements. It comprises global autocorrelation and local autocorrelation, with the Moran index frequently employed as a measure of global autocorrelation, calculated by the formula below equation (1) (8):

(1) $$I=\frac{\overset{n}{\underset{i=1}{\sum }}\overset{n}{\underset{j=1}{\sum }}W_{ij}\left(\mathrm{Xi}-\bar{X}\right)\left(Xj-\bar{X}\right)}{{\left(\frac{1}{n}\overset{n}{\underset{i=1}{\sum }}\mathrm{Xi}-\bar{X}\right)}^{2}\overset{n}{\underset{i=1}{\sum }}\overset{n}{\underset{j=1}{\sum }}W_{ij}}$$

Where I denote the global Moran index, *n* is the number of observation units, Xj is the PM2.5 concentration values of units i and j, Wij denotes the spatial weight between points i and j, Wij equals 1 means that the two are neighboring, Wij equals to 0 means that the two are not neighboring, and $\bar{X}$ is the sample mean value. Moran’s index is between [−1, 1], I > 0 means positive spatial autocorrelation, i.e., the observed attributes are spatially aggregated, I < 0 means negative spatial autocorrelation, i.e., the observed attributes are spatially dispersed, and I = 0 means that the variables are not spatially correlated.

#### 2.3.2 Geographic detector

Geoprobes, a new statistical method used to detect spatial heterogeneity and reveal the driving mechanisms behind it, have been widely used in the analysis of land use driving mechanisms.

To detect the spatial heterogeneity of the dependent variable PM2.5 concentration change Y, and the magnitude of the influence of the driving factor X on the dependent variable PM2.5 concentration change Y, which is measured by the q-value, the expression is calculated as equations (2), (3) (21):

(2) $$q=1-\frac{\sum_{h=1}^{L}N_{h}\sigma_{h}^{2}}{N\sigma_{h}^{2}}=1-\frac{SSW}{SST}$$

(3) $$SSW=\overset{L}{\underset{h=1}{\sum }}N_{h}\sigma_{h}^{2},\;SST=N\sigma^{2}$$

Where q value is the influence of a single factor on land use change, q ∈ [0, 1], the larger the q value, the greater the influence of the driving factor on the change of PM2.5; h = 1, 2, 3 – L; L is the stratification of the factor X; N_h, *N* is the number of samples in a stratum and the whole region, respectively; σ_h^2, σ^2 is the sum of squares of a stratum and the whole region, respectively. sSW and SST are the within-stratum sum of squares (Within Sum of Squares) and the total region-wide sum of squares (Total Sum of Squares), respectively.

## 3 Results and analysis

### 3.1 Temporal variation pattern of PM2.5 concentration

**Figure 3:**
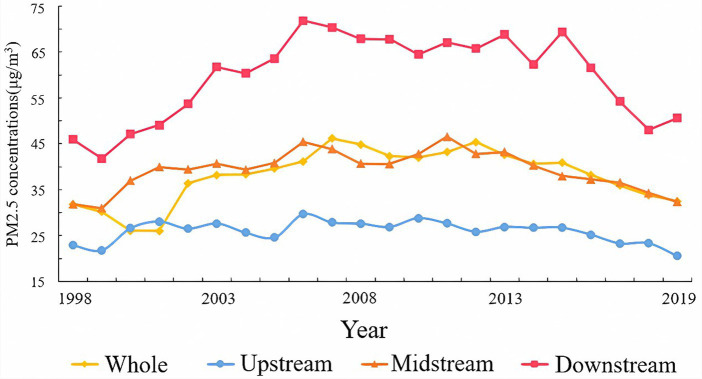
The proportion of different PM2.5 concentration grades in 538 counties in the study area from 1998 to 2019.

This study aims to visually depict the temporal evolution of PM2.5 in the Yellow River Basin from 1998 to 2019. It calculates the average annual PM2.5 concentration for the Yellow River Basin and its sub-district counties, then presents the trend of PM2.5 changes in the Yellow River Basin from 1998 to 2019, as illustrated in Figure 3. The average annual PM2.5 concentration in the Yellow River Basin exhibited an overall trend of first increasing and then decreasing, with a rise from 31.80 μg/m^3^ in 1998 to 32.47 μg/m^3^ in 2019, marking a 0.67 μg/m^3^ increase over the study period. The average annual growth rate was 0.03 μg/m^3^. Given the significant disparities across different phases, the analysis is categorized into three distinct phases:

During the first phase (1998–2006), industrial development led to a significant surge in energy consumption, resulting in a series of environmental challenges amidst economic growth. This period witnessed a continuous rise in PM2.5 concentrations by nearly 30%. However, since 2005, there has been a notable suppression in the annual average PM2.5 concentration, with its peak occurring in 2006. This shift can be attributed to the implementation of various measures aimed at enhancing environmental quality and curbing energy consumption growth by the government. These measures include the enactment of environmental regulations such as the Energy Conservation Law and the Air Pollution Prevention and Control Law, along with intensified supervision and management of pollution sources, particularly industrial enterprises. The enforcement of these laws and policies has effectively reduced industrial emissions of air pollutants like PM2.5, thereby making a significant contribution to mitigating environmental pollution.

During the second stage (2007–2011), there was a gradual decrease in PM2.5 concentration, dropping from 44.87 μg/m^3^ in 2007 to 43.21 μg/m^3^ in 2011, resulting in an annual reduction of 1.66 μg/m^3^. Following a period of rapid economic growth from 2000 to 2006, China implemented a macroeconomic policy characterized by “stability-oriented policies and structural adjustment” in 2007. This shift indicated a move towards prioritizing stable economic growth while simultaneously restructuring the economy. Additionally, China increasingly emphasized the pursuit of “fast and high-quality” economic development. Notably, at the 17th National Congress of the Communist Party of China, the concept of building an ecological civilization was introduced for the first time. This strategic goal aimed to harmonize economic development with environmental protection, emphasizing the importance of aligning human activities with nature. The emphasis on ecological civilization influenced policies and measures aimed at controlling PM2.5 emissions, fostering a regulatory environment conducive to pollution reduction and sustainable development.

During the third stage (2012–2019), PM2.5 concentrations in the Yellow River Basin exhibited overall fluctuating patterns. From 2008 to 2010, influenced by the global financial crisis, the promotion of sustainable development concepts, and subsequent policy implementations emphasizing “resource conservation and environmental friendliness,” PM2.5 concentrations notably decreased. However, with the implementation of the “Four Trillion Yuan Stimulus Plan,” China’s real economy gradually achieved a soft landing, leading to a resurgence in PM2.5 concentrations. In 2013, haze engulfed over 25 provinces and more than 100 large and medium-sized cities in China, severely impacting the country’s economic development, public health, and government image. Nevertheless, due to the vigorous implementation of policy documents such as the “Twelfth Five-Year Plan for Key Regions’ Atmospheric Pollution Prevention and Control” and the “Action Plan for Air Pollution Prevention and Control,” PM2.5 concentrations exhibited a sustained decline. However, in 2017, haze once again blanketed cities, prompting the Ministry of Environmental Protection and the Ministry of Industry and Information Technology to issue the strictest “production suspension orders” in history. Many cities in Shandong, Henan, and Shanxi provinces in the Yellow River Basin were included in these measures. The aggressive implementation of the aforementioned policy documents by the State Council significantly influenced the reduction of PM2.5 concentrations by imposing strict emission control measures on industries and enhancing environmental protection efforts.

**Figure 4:**
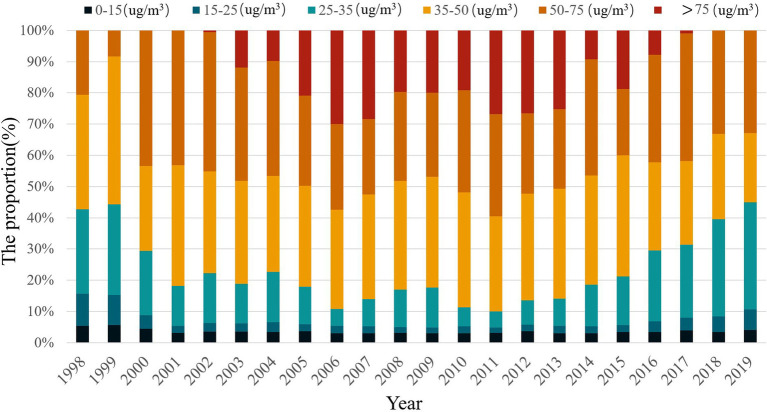
The trend of PM2.5 concentration in the Yellow River Basin from 1998 to 2019.

To further analyze the temporal changes in PM2.5 concentration across 538 counties within the Yellow River Basin region, this study utilizes the annual average limit value of PM2.5 concentration outlined in the Ambient Air Quality Standard (GB3095-2012) issued by the World Health Organization and the Ministry of Environmental Protection of the People’s Republic of China. Following established methodologies in related literature, the annual average PM2.5 concentration is categorized into six intervals for analysis. The distribution of counties within each interval over the study period is illustrated in Figure 4. The findings reveal the following trends: (1) The number of counties in the Yellow River Basin with PM2.5 annual average concentrations below 15 μg/m^3^ is relatively low, exhibiting minor fluctuations over time. The proportion decreased from 9.53% in 1998 to 7.60% in 2019, indicating deteriorating air quality in the region and limited occurrence of areas with low PM2.5 concentrations. (2) The percentage of counties with annual average PM2.5 concentrations ranging from 15 to 25 μg/m^3^ and 25–35 μg/m^3^ increased by 8.98 and 8.56%, respectively, between 1998 and 2019. (3) On average, 6.74% of counties in the Yellow River Basin recorded annual average PM2.5 concentrations exceeding 35 μg/m^3^, with this proportion escalating to over 7.5% during years of severe pollution (e.g., 2010, 2011, and 2013). Among these, the highest number of counties fell within the 5–7.5 μg/m^3^ range, comprising an average proportion of 31.86% relative to the overall level.

### 3.2 Spatial variation trend analysis of PM2.5 concentration

**Figure 5:**
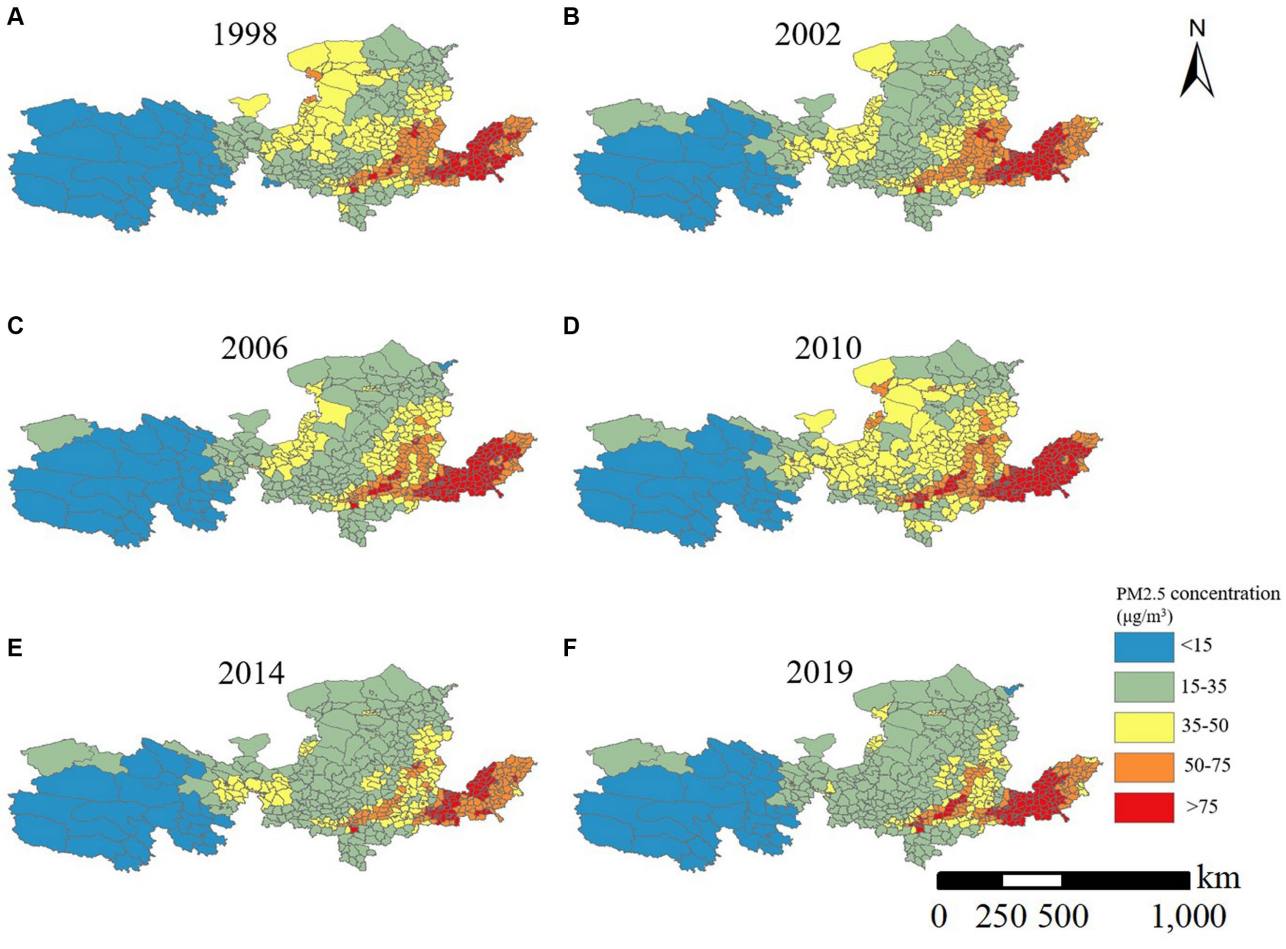
Spatial distribution of PM2.5 concentration in the Yellow River Basin.

To comprehensively examine the spatial variation characteristics of PM2.5 concentration, this study conducts spatial clustering of the mean PM2.5 concentration in the Yellow River Basin across multiple years. Recognizing that visual analysis based on equal intervals may overlook years with significant changes in PM2.5 concentration, we aim to enhance the objectivity and accuracy of our findings. Therefore, we spatially visualize the PM2.5 concentration distribution across counties in the Yellow River Basin. Specifically, we select six representative years—1998, 2002, 2006, 2010, 2014, and 2019—for spatial analysis (refer to Figure 5).

In the Yellow River Basin, areas with low PM2.5 concentrations are primarily situated in less populated and ecologically pristine regions of Qinghai Province (excluding Xining City and Haidong City) and central Inner Mongolia. Conversely, high PM2.5 concentration areas are found at the junction of Shaanxi, Gansu, Ningxia, and Mongolia, known for their abundant coal, oil, and natural gas resources, as well as densely populated regions of Henan and Shandong Provinces, characterized by robust industrial bases. Specifically, in 2000, counties in the Yellow River Basin with PM2.5 concentrations below 3.5 μg/m3 were located in Qinghai Province, Gansu Province (Haibei Mongol Autonomous County and Akhasak Autonomous County), central Inner Mongolia, north-central Shaanxi Province, and Yantai City in Shanxi and Shandong Provinces. However, 63.34% of counties in the Ningxia Hui Autonomous Region and 42.53% of counties in Gansu Province had an annual average PM2.5 concentration exceeding 75 μg/m3. Air pollution in some counties in west-central Henan Province, western Shandong Province, and eastern Qinghai Province (such as Xining City and Haidong City) reached 50–75 μg/m3, while PM2.5 concentrations in Inner Mongolia’s Wuyuan County, Hangjin Banner, and western Ertuoqqi Former Banner, as well as counties in the southern part of Shaanxi Province, exceeded 3.5 μg/m3. Overall, in 2000, the Yellow River Basin formed the “Shaanxi, Gansu, Ningxia, and Mongolia” and “Henan-Shandong” pollution zones.

With the rapid urbanization and industrialization in China during 2000–2011 and the implementation of strategies like the “Western Development,” air pollution in the Yellow River Basin worsened, with PM2.5 pollution escalating notably in Henan and Shandong provinces, and issues in Shaanxi and Shanxi provinces becoming more pronounced. Compared to 2000, counties with PM2.5 annual average concentrations exceeding 75 μg/m^3^ expanded eastward, mainly concentrated in Shanxi, Henan, and Shandong Provinces, with sporadic distribution in Shaanxi Province (Weinan City, Xianyang City, and Xi’an City junction zone). Though air quality improved within the “Ganning” region, the southern part of Gansu Province (including Baiyin City, Dingxi City, Lanzhou City, and Linxia Hui Autonomous Prefecture), Ningxia Hui Autonomous Region (such as Pingluo County in Shizuishan City, Helen County, and Yongning County in Yinchuan City), and Inner Mongolia Autonomous Region (Uda District) still exhibited annual average PM2.5 concentrations exceeding 50 μg/m^3^.

With increased national attention to air pollution and initiatives for ecological civilization construction and sustainable development, the atmospheric quality in the Yellow River Basin significantly improved by 2018, with no counties reporting PM2.5 annual average concentrations exceeding 75 μg/m^3^. Compared to 2011, the number of counties with annual average PM2.5 concentrations below 35 μg/m^3^ increased by 222 counties, a 1.85-fold rise. Besides the improved environmental quality in Qinghai Province and central Inner Mongolia with annual average PM2.5 concentrations below 1.5 μg/m^3^, Gansu Province, Ningxia Hui Autonomous Region, and Shaanxi Province also experienced substantial air quality improvements. However, during the same period, some counties in Henan Province, Shandong Province, south-central Shanxi Province (including Jinzhong, Linfen, and Yuncheng), and Shaanxi Province (including Weinan and Xianyang) still had average annual PM2.5 concentrations exceeding 3.5 μg/m^3^, warranting focused atmospheric control efforts in the Yellow River Basin.

### 3.3 Spatial clustering trends

#### 3.3.1 Global spatial autocorrelation analysis

**Figure 6:**
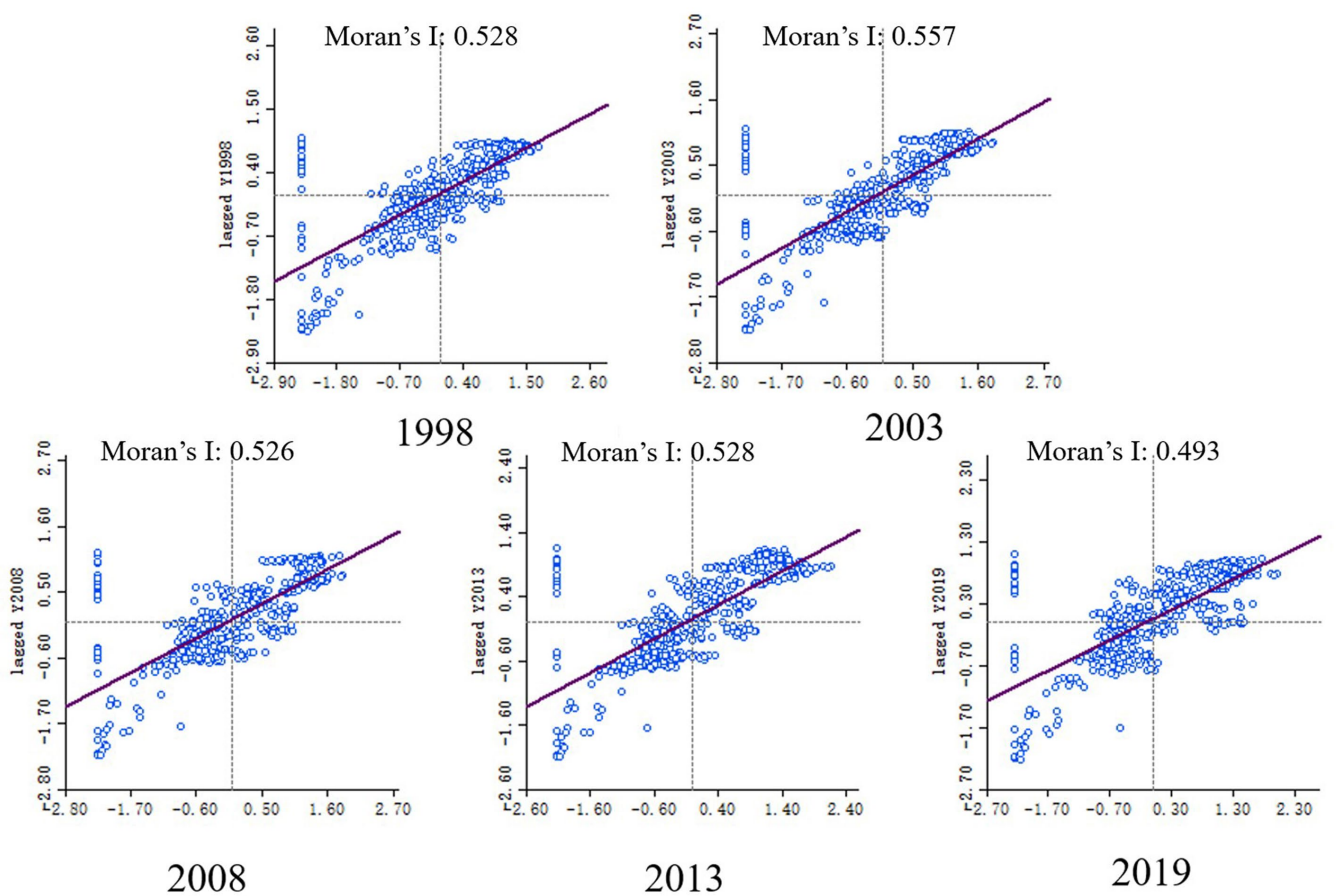
Moran’s I index of PM2.5 for five years from 1998 to 2019.

To analyze the spatial distribution characteristics of PM2.5 concentration in the Yellow River Basin, the remote sensing inversion data of PM2.5 were statistically partitioned at the county level of the administrative unit. Five years of PM2.5 annual average concentration data from 1998, 2003, 2008, 2013, and 2019 were selected for the global spatial autocorrelation analysis of PM2.5 concentration in the Yellow River Basin. Analyzing their Moran’s I index, it was observed (Figure 6) that the Moran’s I index for all 5 years was positive and passed the significance test at 0.01, indicating spatial positive correlation in the distribution of PM2.5 across the Yellow River Basin, suggesting evident spatial clustering characteristics. The global Moran’s I index exhibited fluctuating trends over time, initially increasing from 0.528 in 1998 to 0.557 in 2003, declining to 0.526 in 2008, increasing again to 0.528 in 2013, and then decreasing to 0.493 in 2019. Notably, the index for 2008 surpassed that of 1998, suggesting an increasing spatial correlation of PM2.5 concentration among counties in the Yellow River Basin and an enhancement in spatial agglomeration. This indicates that counties with high PM2.5 concentrations tend to be clustered within specific areas, as do those with low concentrations. The lower Moran’s I index in 2019 compared to previous years suggests a weakening of PM2.5 agglomeration in the Yellow River Basin.

#### 3.3.2 Local spatial autocorrelation analysis

**Figure 7:**
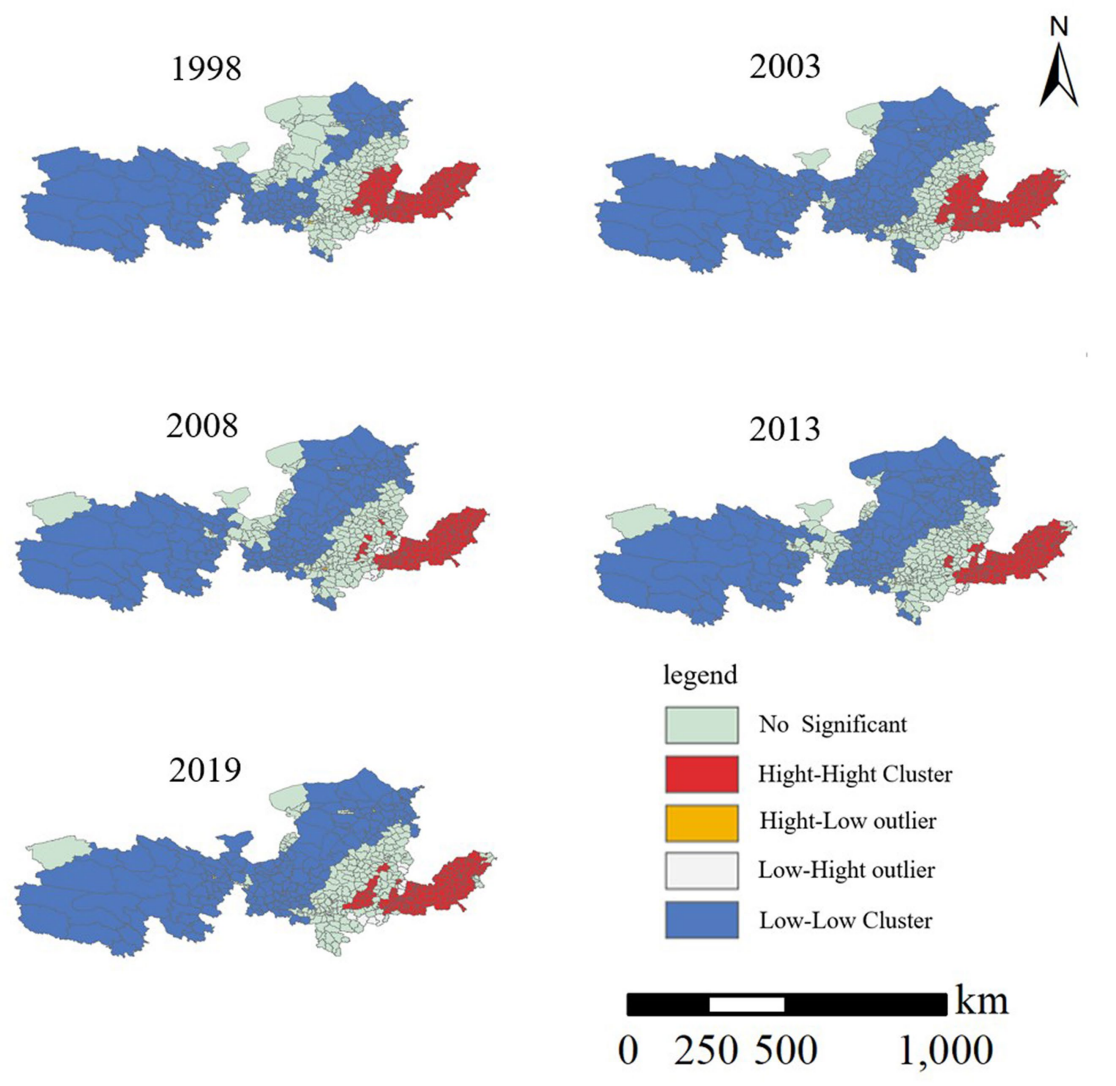
Spatial cluster distribution of PM2.5 mass.

The annual average ρ(PM2.5) exhibits significant clustering of high and low values, demonstrating a spatial club convergence phenomenon (Figure 7). Among these, 33 cities show HH-type clustering, accounting for 37.08%, primarily concentrated downstream of the Yellow River Basin in the Yuzhu area. These regions, undergoing rapid industrialization, are dominated by resource-intensive industries, leading to substantial emissions of air pollutants and persistent high pollution levels. Conversely, 26 cities exhibit LL-type clustering, representing 29.21% of the total, mainly situated in the upper reaches of the Yellow River Basin, including Qinghai, Gansu, Ningxia, and western Inner Mongolia, characterized by good air quality, low economic development, sparse population, and minimal pollution emissions. Thus, stable LL-type agglomeration areas have formed in the Yellow River Basin, with hotspots of severe PM2.5 pollution and coldspots of excellent air quality distributed at two distinct poles, respectively, in the southeast and northwest of the Hu Huanyong line. In 2019, hotspot areas were mainly concentrated in the Central Plains Urban Agglomeration and central and western Shandong Peninsula Urban Agglomeration, while coldspot areas were predominantly distributed in Qinghai, Gansu, Ningxia, and the western part of Inner Mongolia, boasting excellent air quality. This spatial clustering trend is consistent with Figure 7, wherein low-value areas are primarily concentrated in the upper reaches of the Yellow River Basin in Qinghai, Gansu, and Ningxia, while high-value areas are located in the lower reaches of the Yellow River Basin in Henan, and Shandong.

### 3.4 Driver analysis

The determinants of PM2.5 primarily include precipitation, temperature, economic, and policy factors. Economic activities, reflected by luminous light data, are considered in this study. Given the geographical characteristics of the Yellow River Basin, extensive forestry initiatives like the Three-North Protection Forest Construction Project and the Middle Yellow River Protection Forest Construction Project have been implemented. Moreover, national policies promoting afforestation and forest conservation have increased forest cover in the basin. The Normalized Vegetation Index (NDVI) represents vegetation cover. To comprehensively analyze the factors driving PM2.5 concentration changes, panel data from 2003, 2008, 2013, and 2019 comprising 559 districts and counties are selected. Geodetector analysis is employed to detect spatial differentiation features and patterns. Ten key factors, including average air temperature (X1), air pressure (X2), sunshine duration (X3), precipitation (X4), NDVI (X5), population density (X6), secondary industry growth (X7), financial expenditure (X8), night lighting (X9), and electricity consumption (X10), which significantly influence PM2.5, are examined across the Yellow River Basin districts and counties.

#### 3.4.1 Analysis of domain-wide drivers in the Yellow River Basin

**Figure 8:**
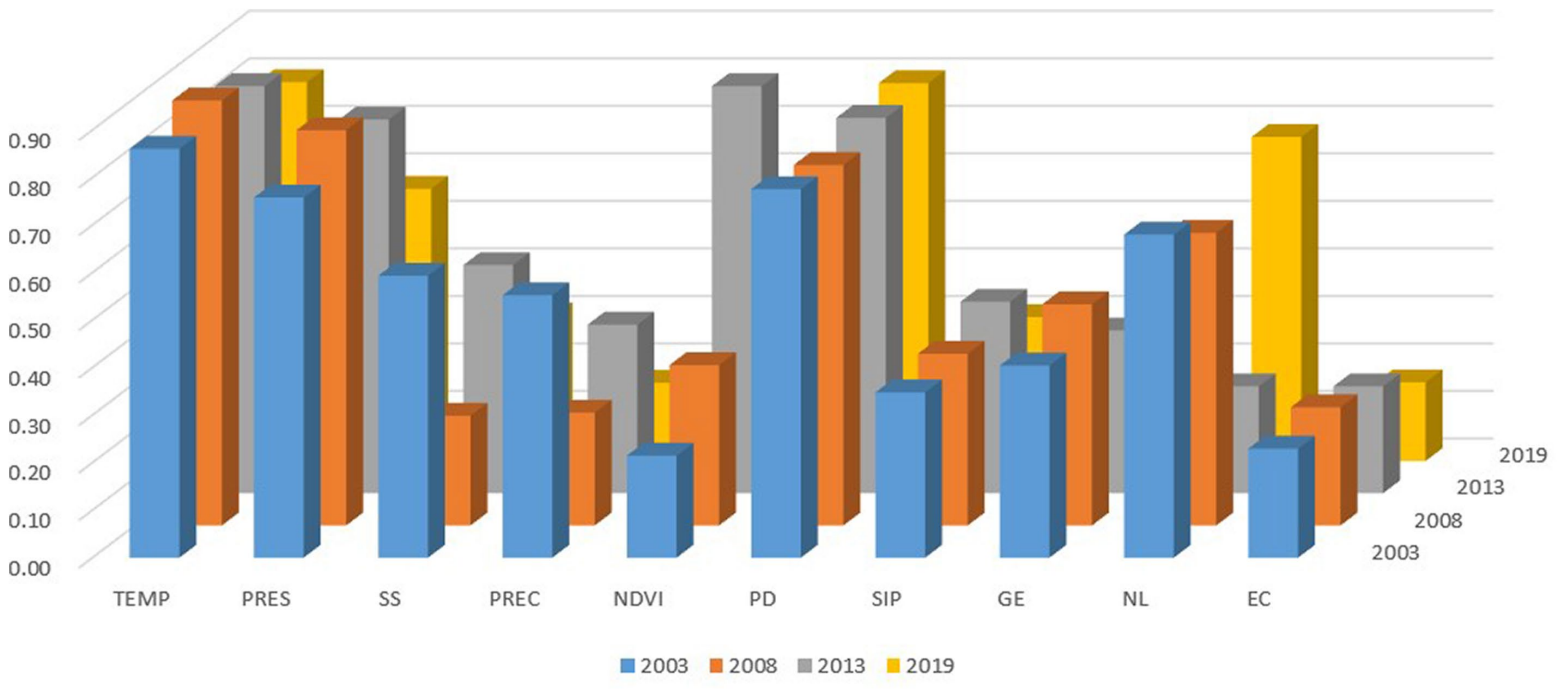
The GeoDetector q-statisticand of socio-economic.

To elucidate the socio-economic factors driving PM2.5 concentration across the entire Yellow River Basin, the explanatory power (q-value) of each socio-economic factor was assessed basin-wide using divergence and factor detectors. The findings are depicted in Figure 8.

As illustrated in Figure 8, mean air temperature, mean air pressure, population density, and nighttime lighting emerge as the four primary factors with the greatest explanatory power for PM2.5 on a basin-wide scale. (1) Mean air temperature consistently exhibits the highest explanatory power exceeding 0.80, highlighting its paramount influence on PM2.5 levels in the Yellow River Basin. (2) Average air pressure shows notable significance in 2003, 2008, and 2013, with an explanatory power of approximately 0.79, ranking second. Situated between the Tibetan Plateau and the North China Plain, the Yellow River Basin’s intricate and low-lying terrain implies that air pressure fluctuations exert a considerable impact on atmospheric conditions. (3) Population density registers high significance, surpassing 0.76, and maintains stability. Geo-detector analyses reveal that PM2.5 pollution hotspots in the Yellow River Basin primarily cluster in densely populated regions such as Henan and Shandong, indicating substantial spatial overlap between heavily polluted zones and areas of high population density. (4) Nighttime lighting exhibited significant explanatory power in 2003 and 2008, approximately 0.65, but experienced a decline in both significance and explanatory power in 2013, only to rise again to 0.68 in 2019. This fluctuation suggests a downturn in nighttime lighting across the Yellow River Basin in 2013 due to certain factors. It is plausible that the issuance of the Measures for Implementing the Regulations on Promoting Air Pollution Prevention and Control in Henan Province by the Henan Provincial Government in 2013, which mandates strict control over industrial and domestic waste pollution of the air environment and stringent management of nighttime lighting in industrial parks and ecological key areas, contributed to this decline.

#### 3.4.2 Analysis of driving factors for different zones

**Figure 9:**
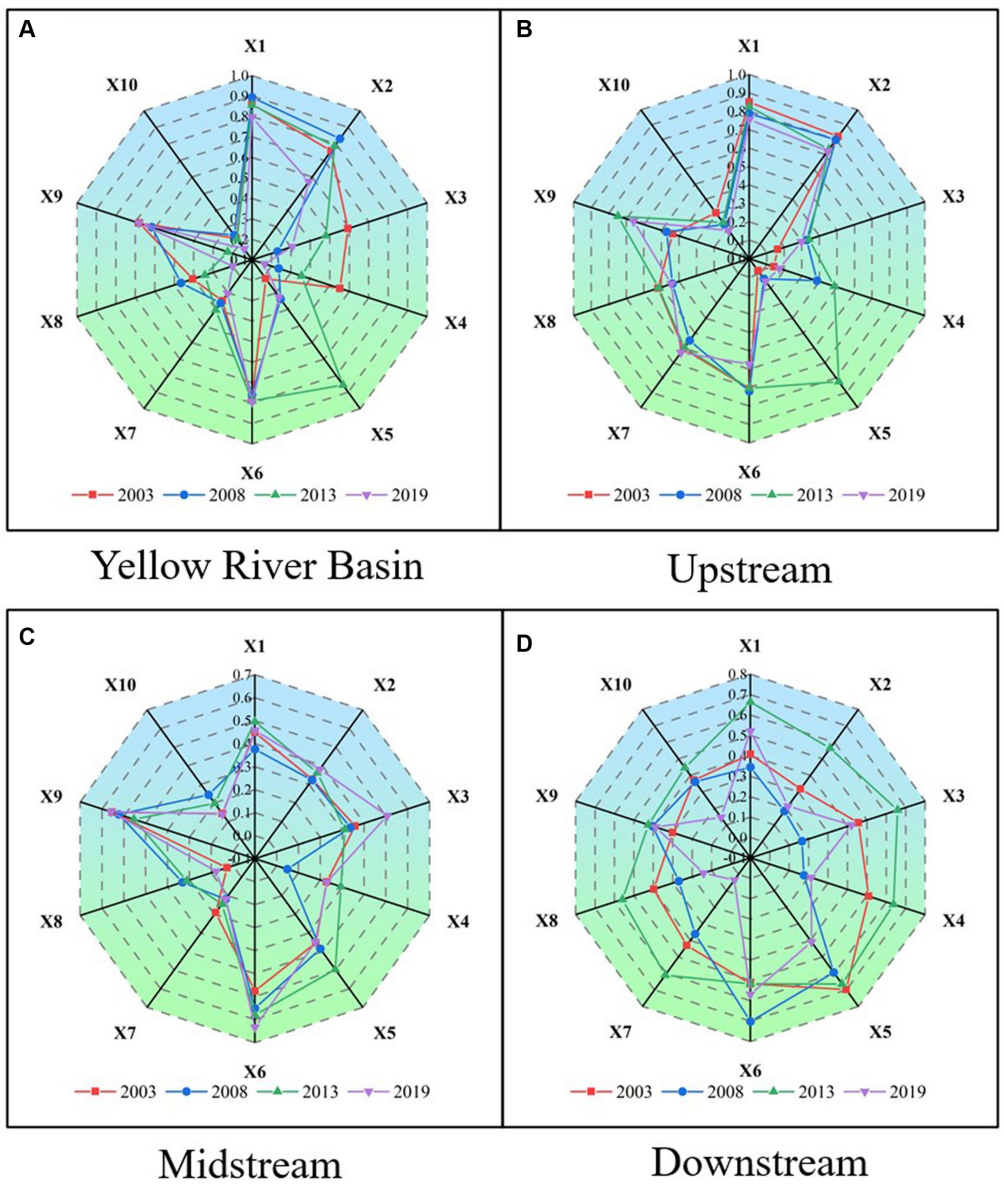
Radar chart of contribution rate of PM2.5 influence factors in different zones.

In this study, a detector was employed to analyze the factors across four periods of data (2003, 2008, 2013, and 2019), partitioning the upper, middle, and lower reaches of the Yellow River Basin to determine the drivers of PM2.5. As depicted in Figure 9, substantial geographic variability in the influencing factors of PM2.5 pollution is evident.

From the perspective of each subregion, the upstream area primarily falls within the low-low aggregation zone of air pollution in the Yellow River Basin. Dominant factors driving the divergence of PM2.5 concentration in these counties include average air temperature (0.85), average air pressure (0.82), and population density (0.71), while electricity consumption (0.22), vegetation cover index (0.30), and annual precipitation (0.30) exhibit lower impact levels. The upstream regions experience greater terrain variation and more complex meteorological conditions. For instance, Qinghai Province gradually slopes from the Tibetan Plateau to the southeast, encompassing diverse terrain such as mountains, plateaus, basins, and hills, with altitudes ranging from 0 to 6,350 meters above sea level. Such topographical and meteorological intricacies result in significant spatial and temporal disparities in surface temperature and humidity, leading to an uneven distribution of temperature across the region. These factors significantly influence pollutant emissions, resulting in substantial spatial variation in emissions between regions. Moreover, the upstream region has relatively small populations and industries, particularly in provinces like Qinghai and Gansu, where ethnic minorities are more prevalent and urbanization levels are lower. This contributes to relatively lower emissions of pollutant particles such as PM2.5 in this region. Generally, enhanced vegetation cover promotes the absorption and deposition of atmospheric particles, while ample precipitation flushes out air pollutants, significantly improving local PM2.5 pollution. The relatively minor impact of vegetation and precipitation on the spatial heterogeneity of PM2.5 in upstream county units may be attributed to the overall balanced and stable annual precipitation and surface vegetation growth in this region, resulting in less discernible differences in their effects on particulate matter emissions. Regarding electricity consumption, it can be inferred that the upstream area’s relatively lower development level, smaller population, and industries result in electricity consumption not being a major contributor to PM2.5 pollution. Although industrial, agricultural, and domestic energy consumption may contribute to PM2.5 pollution to some extent, their influence is smaller compared to downstream areas.

The midstream region generally falls within the high-high concentration zone of air pollution in the Yellow River Basin watershed. Dominant factors driving PM2.5 concentration divergence in counties within this region include population density (0.56) and nighttime lighting (0.52). This area typically experiences higher population densities due to increased urbanization levels in regions characterized by rapid economic development, which is a key driver of PM2.5 concentration disparities. For instance, Zhengzhou City, the capital of Henan Province, serves as a commercial hub and logistical transit point, exhibiting high population density and significant urban sprawl, leading to severe PM2.5 pollution during winter months, often prompting red alerts. Additionally, certain cities in the midstream region of the Yellow River, influenced by heavy industrial activity and intense nighttime lighting, also contribute to elevated PM2.5 concentrations. For instance, Anyang City in Henan Province, situated at the boundary of Henan and Shanxi Provinces, is predominantly characterized by heavy industries such as coal, iron, and steel production, with its elevated PM2.5 levels attributed to factors including industrial composition and air circulation patterns. Similarly, Baoji City in Shaanxi Province, known for industries like aluminum and electricity production, also experiences relatively high PM2.5 concentrations.

The downstream region of the Yellow River Basin generally falls within the high-high aggregation zone of haze pollution. Dominant factors driving the divergence of PM2.5 concentrations in counties within this region include vegetation cover index (0.70), population density (0.52), and precipitation (0.51). The lower reaches of the Yellow River feature predominantly flat terrain and sparse vegetation cover, leading to elevated concentrations of PM2.5 in the air. For instance, cities like Linyi and Zibo, characterized by low vegetation cover, exhibit medium to high PM2.5 concentrations. Moreover, higher population density and increased traffic congestion are significant contributors to PM2.5 concentration disparities in this region. Take Linyi City as an example, with its relatively high population density and congested traffic conditions, resulting in elevated PM2.5 levels. Additionally, the lower reaches of the Yellow River experience limited precipitation, making them susceptible to droughts and dusty weather, which hampers air diffusion and exacerbates PM2.5 concentrations. Therefore, strategies such as urban greening initiatives, enhanced vegetation coverage, traffic congestion alleviation, adoption of clean energy, and industrial pollution control are essential for mitigating PM2.5 concentrations in the region. Furthermore, proactive measures to prevent dusty weather and preserve ecological balance are crucial steps in reducing PM2.5 pollution.

## 4 Discussion

This study examines the spatial and temporal distribution patterns of PM2.5 in the Yellow River Basin and its subregions while investigating the impact of natural and social factors on PM2.5 levels. Existing studies primarily concentrate on municipal and higher administrative divisions (30, 36), with limited research conducted at the county level. This investigation reveals that the proportion of counties with annual average PM2.5 concentrations below 15 μg/m^3^ in the Yellow River Basin decreased from 9.53% in 1998 to 7.60% in 2019, indicating deteriorating air quality in the region and the challenge of achieving widespread low PM2.5 areas. This deterioration can be attributed to rapid industrialization and urbanization in the Yellow River Basin over recent decades, resulting in significant industrial and transportation emissions negatively impacting air quality. During the process of economic development, many countries may have prioritized economic growth over environmental protection and air quality improvement.

The PM2.5 concentration in the Yellow River Basin, characterized by its complex geography, is influenced by various factors. Population densities in the upstream, midstream, and downstream regions of the Yellow River Basin are significant, suggesting that human activities contribute to higher PM2.5 concentrations. Compared to previous studies on the Yellow River Basin as a whole (21, 30, 36), the main drivers of the middle reaches remain consistent, while those of the upper and lower reaches have shifted. In the upstream, barometric pressure emerges as a key driver of PM2.5. Air pressure, a crucial component of atmospheric circulation, affects atmospheric movement, stability, and consequently, air quality. Variations in specific air pressure systems in the upper reaches influence air stability, affecting PM2.5 dispersion and accumulation. Moreover, changes in air pressure impact meteorological conditions such as precipitation distribution and wind patterns, further affecting air quality. Additionally, the higher elevation of upstream areas amplifies the impact of topography on barometric pressure, making it a prominent driving factor. Mutual interactions between the middle reaches of the Yellow River Basin and the overall basin may result in consistent drivers. For instance, the ecological conditions in the midstream region may influence the basin’s ecological balance, aligning the drivers. In the downstream, NDVI and precipitation emerge as primary drivers of PM2.5 due to its flat terrain, conducive to water retention and vegetation growth. Conversely, the mountainous terrain in the upper and middle reaches limits vegetation cover, making topography less influential. Moreover, the downstream’s humid climate and high precipitation further enhance vegetation growth, making precipitation a significant driver. Conversely, arid climates in the upper and middle reaches diminish the impact of precipitation.

This study possesses several notable features compared to previous research. Firstly, we conducted a comprehensive examination of the spatial and temporal evolution, as well as the driving mechanisms, of PM2.5 concentration in the Yellow River Basin at the county scale—a novel approach that addresses previous research gaps. Secondly, we categorized the Yellow River Basin into upper, middle, and lower reaches, conducting in-depth analyses of the driving mechanisms within each subregion. This approach enhanced the study’s precision and comprehensiveness.

Based on the analysis of PM2.5’s spatial and temporal characteristics and its driving factors in the Yellow River Basin, the following environmental management initiatives are proposed: (1) Enhanced Monitoring and Assessment: Establishing a comprehensive environmental monitoring network prioritizing air quality monitoring at the county level. Regular assessments of PM2.5 concentration trends will furnish a scientific basis for formulating targeted management measures. (2) Adapted Management Strategies: Acknowledging the variability of PM2.5 concentration driving mechanisms across subregions within the Yellow River Basin, management strategies should be customized to local conditions. To enhance air quality in the upstream region, initiatives such as implementing a robust multi-level pollutant emission reduction system, enhancing ecological protection and resource management, and promoting cleaner production and energy restructuring are recommended. (3) Source Control and Clean Energy Promotion: Addressing pollution issues in the middle reaches of the Yellow River Basin necessitates controlling pollution emissions at their source. This entails advocating for and popularizing clean energy solutions, and strengthening motor vehicle tailpipe emission controls to efficiently manage pollution sources and mitigate pollutant emissions, thus improving PM2.5 concentration levels in the region. (4) Integrated Ecological Governance and Urbanization Development: To combat PM2.5 pollution in the middle reaches of the Yellow River Basin, a fusion of ecological governance and urbanization development is advocated. This involves ramping up investment in ecological and environmental protection projects, augmenting vegetation coverage and groundwater levels, fostering the principles of sustainable development, promoting agricultural modernization and green development, fortifying urban planning and management, alleviating urban traffic congestion, enhancing the urban environment, and ultimately enhancing regional air quality.

## 5 Conclusion

As the primary pollutant contributing to haze pollution, PM2.5 significantly hampers the progress of ecological civilization construction and high-quality development in the Yellow River Basin. This study utilizes annual average concentration data of PM2.5 from 1998 to 2019 to analyze spatial and temporal patterns within the basin and its subregions. Employing detectors, we investigate the driving mechanisms of PM2.5 in the Yellow River Basin during four time intervals: 2003, 2008, 2013, and 2019. The main findings are outlined below.

1. The annual average PM2.5 concentration in the Yellow River Basin from 1998 to 2019 exhibits a fluctuating trend, characterized by periods of increase followed by decrease. Specifically, the period spanning 1998–2007 witnessed a rapid rise in PM2.5 concentration. Subsequently, from 2008 to 2011, there was a gradual decline in PM2.5 concentration. This downward trend continued from 2012 to 2019.
2. The spatial autocorrelation analysis reveals significant spatial differentiation and agglomeration tendencies of PM2.5 pollution across the Yellow River Basin, characterized by spatial club convergence. Specifically, high-high (HH) clusters are predominantly found in the downstream regions of Yu and Lu, while low-low (LL) clusters are concentrated in the upstream areas encompassing Qinghai, Gansu, Ningxia, Mengxi, and northern Shaanxi.
3. The geo-detector analysis indicates that average air temperature, population density, and nighttime lighting emerge as the most influential factors across the entire basin, exhibiting strong explanatory power and significant impacts on PM2.5 pollution. Additionally, for the Yellow River Basin overall, the secondary industry’s added value and financial expenditure demonstrate consistent strength and positive correlations with air pollution levels.
4. For various subregions of the Yellow River Basin, PM2.5 pollution in the upper reaches is primarily driven by air temperature, air pressure, and population density. In the middle reaches, population density, and nighttime lighting index emerge as the main drivers of PM2.5 pollution, attributable to heightened human activities. Downstream, PM2.5 pollution is chiefly influenced by vegetation cover index, precipitation, and population density.

## Data availability statement

The raw data supporting the conclusions of this article will be made available by the authors, without undue reservation.

## Author contributions

LH: Conceptualization, Data curation, Funding acquisition, Project administration, Resources, Supervision, Writing – review & editing. MH: Conceptualization, Formal analysis, Investigation, Methodology, Visualization, Writing – original draft. YW: Formal analysis, Investigation, Methodology, Writing – original draft. HW: Funding acquisition, Resources, Supervision, Visualization, Writing – review & editing. JN: Formal analysis, Funding acquisition, Validation, Writing – review & editing.

## Footnotes

^1^https://sites.wustl.edu/acag/datasets/surface-pm2-5

^2^http://www.resdc.cn

^3^http://www.geodata.cn

^4^https://modis.gsfc.nasa.gov/

^5^https://landscan.ornl.gov/

^6^https://data.tpdc.ac.cn/

^7^https://www.nature.com/articles/s41597-022-01322-5

## References

[1] Han LJ Zhou WQ Li WF. Impact of urbanization level on urban air quality: a case of fine particles (PM2.5) in Chinese cities. Environ Pollut. (2014) 194:163–70. doi: 10.1016/j.envpol.2014.07.022, PMID: 25113968

[2] Liang Z Wang W Wang Y. Urbanization, ambient air pollution, and prevalence of chronic kidney disease: a nationwide cross-sectional study. Environ Int. (2021) 156:106752. doi: 10.1016/j.envint.2021.106752 34256301

[3] Kim HS Huh JB Hopke PK. Characteristics of the major chemical constituents of PM2. 5 and smog events in Seoul, Korea in 2003 and 2004. Atmos Environ. (2007) 41:6762–70. doi: 10.1016/j.atmosenv.2007.04.060

[4] Lin H Guo Y Kowal P. Exposure to air pollution and tobacco smoking and their combined effects on depression in six low-and middle-income countries. Br J Psychiatry. (2017) 211:157–62. doi: 10.1192/bjp.bp.117.202325, PMID: 28798061 PMC5579326

[5] Zhang YL Cao F. Fine particulate matter (PM2.5) in China at a city level. Sci Rep. (2015) 5:14884. doi: 10.1038/srep14884, PMID: 26469995 PMC4606739

[6] Cesari D Donateo A Conte M. An inter-comparison of PM2.5 at urban and urban background sites: chemical characterization and source apportionment. Atmos Res. (2016) 174-175:106–19. doi: 10.1016/j.atmosres.2016.02.004

[7] Feng S Gao D Liao F. The health effects of ambient PM2.5 and potential mechanisms. Ecotoxicol Environ Saf. (2016) 128:67–74. doi: 10.1016/j.ecoenv.2016.01.030 26896893

[8] Xie Y Dai H Dong H Hanaoka T Masui T. Economic impacts from PM2.5 pollution-related health effects in China: a provincial-level analysis. Environ Sci Technol. (2016) 50:4836–43. doi: 10.1021/acs.est.5b05576, PMID: 27063584

[9] Lin G Fu J Jiang D Hu W Dong D Huang Y. Spatio-temporal variation of PM2.5 concentrations and their relationship with geographic and socioeconomic factors in China. Int J Environ Res Public Health. (2014) 11:173–86. doi: 10.3390/ijerph110100173 PMC3924439 24362546

[10] He C Hong S Mu H Tu P Yang L Ke B. Characteristics and meteorological factors of severe haze pollution in China. Adv Meteorol. (2021) 2021:1–15. doi: 10.1155/2021/6680564

[11] Shen Y Zhang L Fang X. Spatiotemporal patterns of recent PM 2.5 concentrations over typical urban agglomerations in China. Sci Total Environ. (2019) 655:13–26. doi: 10.1016/j.scitotenv.2018.11.105 30469058

[12] Yan J-W Tao F Zhang S-Q Lin S Zhou T. Spatiotemporal distribution characteristics and driving forces of PM2.5 in three urban agglomerations of the Yangtze River Economic Belt. Int J Environ Res Public Health. (2021) 18:2222. doi: 10.3390/ijerph18052222, PMID: 33668193 PMC7967664

[13] Liu X Zou B Feng H. Anthropogenic factors of PM2.5 distributions in China's major urban agglomerations: a spatial-temporal analysis. J Clean Prod. (2020) 264:121709. doi: 10.1016/j.jclepro.2020.121709

[14] Xu X Zhang T. Spatial-temporal variability of PM2.5 air quality in Beijing, China during 2013–2018. J Environ Manag. (2020) 262:110263. doi: 10.1016/j.jenvman.2020.110263, PMID: 32250779

[15] Gu J Bai Z Liu A Wu L Xie Y Li W. Characterization of atmospheric organic carbon and element carbon of PM2.5 and Pm10 at Tianjin, China. Aerosol Air Qual Res. (2010) 10:167–76. doi: 10.4209/aaqr.2009.12.0080

[16] Huang Y Ji Y Zhu Z Zhang T Gong W Xia X. Satellite-based spatiotemporal trends of ambient PM2.5 concentrations and influential factors in Hubei, Central China. Atmos Res. (2020) 241:104929. doi: 10.1016/j.atmosres.2020.104929

[17] Zhang T Liu P Sun X Zhang C Wang M Xu J. Application of an advanced spatiotemporal model for PM2.5 prediction in Jiangsu Province, China. Chemosphere. (2020) 246:125563. doi: 10.1016/j.chemosphere.2019.125563 31884232

[18] Chen W Ran H Cao X. Estimating PM2.5 with high-resolution 1-km AOD data and an improved machine learning model over Shenzhen, China. Sci Total Environ. (2020) 746:141093. doi: 10.1016/j.scitotenv.2020.141093, PMID: 32771757

[19] Wang W Wang N Gao YJ Zuo R Ma SL Wang JJ. Characterization of PM2.5 pollution during heavy pollution in winter and spring in Zhengzhou in 2019. Journal of environmental. Science. (2020) 40:1594–603. doi: 10.13671/j.hjkxxb.2020.0065

[20] Tian L Hou W Chen J Chen C Pan X. Spatiotemporal changes in PM2.5 and their relationships with land-use and people in Hangzhou. Int J Environ Res Public Health. (2018) 15:2192. doi: 10.3390/ijerph15102192, PMID: 30297620 PMC6211054

[21] Li QF Wang Y Ding X. Study on spatial and temporal distribution of PM10 and PM2.5 in Shijiazhuang City based on GIS. China Environ Monit. (2020) 36:173–83. doi: 10.19316/j.issn.1002-6002.2020.02.20

[22] Wang LL Liu XJ Li D Sun YQ. Geographic detection of spatial heterogeneity and drivers of PM2.5 in the Yangtze River economic zone. Environmental. Science. (2022) 43:1190–200. doi: 10.13227/j.hjkx.202106113 35258183

[23] Pang QH Xiang M Zhou WM. Spatial and temporal evolution characteristics of PM2.5 in the Huaihe River ecological and economic zone. Resour Ind. (2022) 24:55–64. doi: 10.13776/j.cnki.resourcesindustries.20211221.013

[24] Xu Y Li XY Huang WT Guo ZD Pan YC Zheng ZW. Spatial and temporal variations of PM2.5 in typical economic zones of China and its relationship with vegetation landscape pattern from 2000 to 2020. Environ Sci. (2023) 44:1852–64. doi: 10.13227/j.hjkx.202205283 37040936

[25] Peng J Chen S Lü H Liu Y Wu J. Spatiotemporal patterns of remotely sensed PM2.5 concentration in China from 1999 to 2011. Remote Sens Environ. (2016) 174:109–21. doi: 10.1016/j.rse.2015.12.008

[26] Wang H Zhang MC Niu JQ Zheng XY. Spatiotemporal characteristic analysis of PM2. 5 in Central China and modeling of driving factors based on MGWR: a case study of Henan Province. Front Public Health. (2023) 11:1295468. doi: 10.3389/fpubh.2023.1295468, PMID: 38115845 PMC10728471

[27] Shi Y Matsunaga T Yamaguchi Y Li Z Gu X Chen X. Long-term trends and spatial patterns of satellite-retrieved PM2.5 concentrations in south and Southeast Asia from 1999 to 2014. Sci Total Environ. (2018) 615:177–86. doi: 10.1016/j.scitotenv.2017.09.241 28968579

[28] Zhang DH Zhou CS He BJ. Spatial and temporal heterogeneity of urban land area and PM2. 5 concentration in China. Urban Clim. (2022) 45:101268. doi: 10.1016/j.uclim.2022.101268

[29] Chen AZ. Temporal distribution characteristics of PM2.5 in Beijing and its influential factors. IOP Conf Ser Earth Environ Sci. (2020) 585:012041. doi: 10.1088/1755-1315/585/1/012041

[30] Chen SQ Zhang H Qi Y. Spatial spillover effects and influencing factors of haze pollution in the Yellow River Basin. Econ Geogr. (2015) 40:40–8. doi: 10.15957/j.cnki.jjdl.2020.05.005

[31] Sun J Dang Y Zhu X. A grey spatiotemporal incidence model with application to factors causing air pollution. Sci Total Environ. (2020) 759:143576. doi: 10.1016/j.scitotenv.2020.143576, PMID: 33272599

[32] Teng TW Chen DH Hu F. Evolution of spatial pattern and influencing factors of air pollution in the Yellow River Basin. Geoscience. (2021) 41:1852–61. doi: 10.13249/j.cnki.sgs.2021.10.017

[33] Luo J Du P Samat A Xia J Che M Xue Z. Spatiotemporal pattern of PM2.5 concentrations in mainland China and analysis of its influencing factorsmusing geographically weighted regression. Sci Rep. (2017) 7:40607. doi: 10.1038/srep40607, PMID: 28079138 PMC5228184

[34] Hao Y Liu Y-M. The influential factors of urban PM2.5 concentrations in China: a spatial econometric analysis. J Clean Prod. (2016) 112:1443–53. doi: 10.1016/j.jclepro.2015.05.005

[35] Zhou L Zhou C Yang F Che L Wang B Sun D. Spatio-temporal evolution and the influencing factors of PM2.5 in China between 2000 and 2015. J Geogr Sci. (2019) 29:253–70. doi: 10.1007/s11442-019-1595-0

[36] Wang H Wan QN Huang W Niu JQ. Spatial heterogeneity characteristics and driving mechanism of land use change in Henan Province. Geocarto Int. (2023) 38:2271442. doi: 10.1080/10106049.2023.2271442

[37] Wang JF Xu CD. Geodetector: principle and prospective. Acta Geogr Sin. (2017) 72:116–34. doi: 10.11821/dlxb201701010

[38] Jin FJ Lin YH Ma L Chen Z. Evolution of strategic position and direction of high-quality development in the Yellow River Basin. J Lanzhou Univ. (2022) 50:1–12. doi: 10.13885/j.issn.1000-2804.2022.01.001

[39] Li H Han Y. Characterization of the spatial and temporal evolution of PM2.5 in the Yellow River Basin and analysis of its influencing factors. World Geogr Res. (2022) 31:130–41.

[40] Geng JC Shen S Cheng CX. Spatial and temporal distribution pattern of PM2.5 in the Yellow River Basin during the "13th five-year plan" period and analysis of multi-scale socioeconomic impact mechanisms. J Geo-Inf Sci. (2022) 24:1163–75. doi: 10.12082/dqxxkx.2022.210534

[41] Hammer MS van Donkelaar A Li C Lyapustin A Sayer AM Hsu NC. Global estimates and long-term trends of fine particulate matter concentrations (1998–2018). Environ Sci Technol. (2020) 54:7879–90. doi: 10.1021/acs.est.0c01764, PMID: 32491847

[42] Zhao AZ Xiang KZ Liu XF Zhang XR. Spatio-temporal evolutionpatterns of PM2.5 and relationship with urban expansion in Beijing Tianjin-Hebei urban agglomeration from 2000 to 2018. Environ Sci. (2022) 5:2274–83. doi: 10.13227/j.hjkx.202109226

[43] van Donkelaar A Martin RV Li C Burnett RT. Regional estimates of chemical composition of fine particulate matter using a combined geoscience-statistical method with information from satellites, models, and monitors. Environ Sci Technol. (2019) 53:2595–611. doi: 10.1021/acs.est.8b06392, PMID: 30698001

